# Upper alimentary tract cancer in Natal Indians with special reference to the betel-chewing habit.

**DOI:** 10.1038/bjc.1969.83

**Published:** 1969-12

**Authors:** M. Schonland, E. Bradshaw


					
670

UPPER ALIMENTARY TRACT CANCER IN NATAL INDIANS
WITH SPECIAL REFERENCE TO THE BETEL-CHEWING HABIT

MARY SCHONLAND AND EVELYN BRADSHAW
From the Department of Pathology, Durban Medical School,

University of Natal, Durban, Natal
Received for publication July 31, 1969

THE 1960 Population Census for South Africa indicated that there were 477,125
Indians in South Africa. The Durban region contained 237,344 of these (approxi-
mately 50%). The remainder are scattered through the province of Natal and,
to a lesser extent, the provinces of Transvaal and the Cape. The growth rate for
Indians is regarded by the S.A. Bureau of Statistics as being 2-87% per annum,
and the 1969 S.A. Indian population is therefore approximately 600,000, of whom
about half live in the Durban region (see Table I).

General features

Indians have participated in South African life for 110 years. Between 1860
and 1866 over 6000 Indians were brought to Natal as indentured labourers to
work on sugar-cane farms, and for the ensuing 50 years they were predominantly
engaged in agriculture. In the last 50 years Indians have become increasingly
urbanised and there has been a relative decline in their engagement in agriculture,
as the fields of commerce and industry were entered. In 1960, 80% of those who
were gainfully ernployed were engaged in manufacturing, services or commerce,
while only 12% worked in the agricultural sphere (McCrystal and Maasdorp,
1967).

The Indian population is largely urban dwelling. Only 17% were resident in
rural areas in 1960. Indian household incomes are relatively low, and a substan-
tial amount of poverty exists in the community. In Natal, in 1960, 82 7% of
Indian workers earned less than 800 Rand per annum* (McCrystal and Maasdorp,
1967).

The two main religions practised by Indians are Hinduism and the Islamic
religion. A small proportion of Hindus have turned to Christianity. The
regional variation of religious denominations is shown in Table I. Hindus predom-
inate in Durban, in Natal, and in the whole Indian population, and studies based
on Durban Indians are representative for Natal province. There are relatively
more Moslems in the Cape and Transvaal provinces, and Christians form a larger
proportion of the group in the Cape than elsewhere.

Cancer Incidence

A survey designed to estimate the cancer incidence in South African Indians
was initiated in Durban in 1964. The results of the first three years were published
in 1968 (Schonland and Bradshaw, 1968). As the total number of cases was small,

* In 1960, R2 = ?1.

ALIMENTARY TRACT CANCER AND BETEL CHEWING                        671

TABLE I.-Population Distribution and Religious Groups of South

African Indians, 1960*

Provinces

Durban  _                             South
region    Natal    Cape    Transvaal  Africa

Population .   .    . 237,344 . 394,854 . 18,477 . 63,787  477,125**
% Total population  .  50     . 83      .  4     . 13     . 100

Moslem%    .   .    .   150   . 15-7    . 47-8   . 54 9    . 22-2
Hindu %    .   .    .   77-4  . 77-1    . 280    . 388     . 701
Christian %    .    .   7 6   .   7 2   . 242    .   6 3   .  7-7

* 1960 Population Census.

** Seven Indians live in the Orange Free State.

the investigation was continued for two further years, but further calculations
based on the 5 year investigation produced very little variation from the findings
of the 3 year study.

There are considerable differences between male and female Indians with
regard to upper alimentary tract cancer. The incidence is much greater in Indian
females than males and than that in English of both sexes. A discussion of this
difference now follows, and for convenience, the relevant specific sites are shown
in Table II.

TABLE II.-Age-adjusted Cancer Incidence Rates for Site,s of Mouth,

Pharynx and Alimentary Tract: Indian and English*

Females             Males

Indian   English  Indian    English
A. Upper alimentary tract  .  .  561       17-1     345      34 6
B. Lower alimentary tract  .  .  282     29-6      20 6     37 6
C. Rest of mouth    .    .   .    04       1.7       1.5      3 6
A. Tongue .    .    .    .   .    30       0 6       1.5      1.5

Buccalcavity .   .    .    .   8 1      0 6       4.4      1-7
Oralpharynx .    .    .    .   2K1       1.1      1.0      241
Oesophagus  .    .    .    .  129       2 4       5.5      4.5
Stomach     .    .    .    .  30-0     12.4      22-1     24-8

56-1     17*1      34-5     34-6

B. Smallandlargebowel    .   .    76      14 9       3 6     14 5

Rectum .    .    .    .    .  10-4       78       35       13-1
Liver, biliary tract .  .  .   6 9      20       11-4      2 2
Pancreas and other  .   .      3.3      4.9       2-1      7*8

28-2     29-6      20*6     37-6

C. Lip                            00       0-3       058      2-0

Salivaryglands   .    .    .   04        1-2      0-7      1.1
Nasopharynx .    .    .    .   00 0*2             0-0      0-5

0-4       1-7      1.5      3 6

* Annual Incidence per 100,000 population, standardised to Standard World
Population. Durban Indians, 1964-66; England, 4 regions, 1960-62.

The term " Upper Alimentary Tract " as used here may be defined as including
cancers of the tongue, buccal cavity, oral pharynx, oesophagus and stomach.
The " Lower Alimentary Tract " may be defined as including cancers of the small
bowel, colon, rectum, liver, biliary system and pancreas. The " Rest of Mouth "
may be defined as including cancers of the lip, salivary glands and nasopharynx.

MARY SCHONLAND AND EVELYN BRADSHAW

In the sites grouped under " Upper Alimentary Tract ", English and Indian
males have roughly the same incidence at all specific sites but when comparing
Indian and English females a great difference is observed, the Indian female having
higher rates at all sites. The Indian female develops tongue cancer five times as
often, buccal cancer thirteen times as often, oesophageal cancer six times as often,
and stomach cancer more than twice as often as the English female.

There is little difference between the sexes and the races in the sites grouped
under the title " Rest of Mouth ", English males having the highest rate, due
mainly to lip cancer. In the sites grouped under " Lower Alimentary Tract ",
the English have higher rates for colonic and rectal carcinomas and lower rates
for liver cancers than Indians do.

The undue frequency of cancer of the upper alimentary tract in Indian females
has led us to study the habit of betel-chewing in Durban Indians.

A hospital survey indicated that the habit of betel-chewing was much more
prevalent among Indian females than males. A more comprehensive survey
of the habit among the general Indian population was therefore undertaken with
the assistance of the Institute of Social Research. Correct sociological sampling
techniques were used to define a representative distribution of households through-
out Durban which were studied. A considerable amount of data was acquired,
the findings of which are given below.

The Betel-Chewing Habit Among Durban Indians

Method of survey.- 500 households were studied, which contained 659 families,
or 3678 people. Although the representative distribution of households was
maintained throughout, there was an adjustment in relation to Moslem households,
in an effort to increase the numbers of Moslems interviewed above the statistical
figure of 15%, in order to produce a numerically larger sample of Moslems. There-
fore of the 500 households, 377 were Hindu (2802 people with an average of 5-57
members per family) and 123 were Moslem (876 people, with an average of 5-62
members per family). The findings of the Survey follow.

General Incidence of Chewing in the Indian Population
(a) Age and sex

More female Indians chewed betel than male Indians and this difference
was significant. Of 1842 females of all ages, 30-7 % were chewers, while of 1836
males, 5*5 % were chewers. The percentage of chewers increased with age in
both male and female groups.  Thus 10*3% of males and 71.9% of females, in
the age-group 60 years or more, chewed betel.

Table A of the Appendix shows that the percentage of male and female chewers
in each age-group increases with age, and also that there are far more female
chewers than male chewers at each age level.
(b) Schooling and ability to speak English

Male Indians were better schooled and more fluent in English than female
Indians, and the younger age-groups were better schooled and more fluent in
English than the older age-groups. This is shown in Table B of the Appendix.
If schooling attainments and ability to speak English are regarded as indices of

672

ALIMENTARY TRACT CANCER AND IBETEL CHEWING

westernisation, then Indian males are more westernised then Indian females.
These differences were significant.

Attendance at school and fluency in English was associated with less chewing,
and the converse was also true. Comparisons of schooling and English fluency
were made on those who were 20 or more years old (Appendix Table A). In
females, after age-adjustment to exclude the influence of age per se, schooling and
English fluency produced a significant difference in that there were fewer chewers
in those who had higher schooling attainment and were more fluent in English.
In males no significant difference was obtained for schooling and English fluency
once age had been adjusted.

The tendency towards greater westernisation in Indian males may be one of
the main factors why betel-chewing is less common in males than females in
Durban. It is possible that betel-chewing is a habit which is dying slowly in a
community that is separated from its Asian origin, and becoming westernised, and
that the process of westernisation in the Indian male sex has been accelerated by
entrance into the sphere of western economic activity. Very few Indian females
are economically active.

(c) Moslem and Hindu comparisons

Comparisons between Moslems and Hindus showed no differences as to age,
schooling, or fluency in English, in either sex. Slightly more Moslems were found
to be betel-chewers but there was no significance except in the case of males under
20 years old. As comparisons between chewers were made on adults age 20 years
or more, it was possible to group Moslems and Hindus together for further analyses.

Betel-Chewers: Factors Relating to the Habit in Chewers Aged 20

Years or More

There were 77 male betel-chewers aged 20 years or more (54 Hindu and 23
Moslem males), and 479 female betel-chewers aged 20 years or more (363 Hindu
and 116 Moslem females). There was no significant difference between the age
distributions of Hindu and Moslem male chewers over 20 years old, nor between
the age distribution of Hindu and Moslem female chewers over 20 years old. There
was also no significant difference between the age distributions of all male chewers
when compared with all female chewers. Therefore no age adjustments were
required when testing for differences within the sexes, or between the sexes, in
the over 20 years old groups.

Age first started

The age at which chewing of betel was adopted in male and female Moslems
and Hindus is given in Table 111(a).

Although the mean age at which chewing started was between 20 and 24 years
in all four groups, more Moslem male and female chewers began the habit under
the age of 20 years than did Hindu males and females. Female chewers started
earlier than male chewers in each religious group. These differences are not signi-
ficant. It can be seen from Table III(b) that about two fifths of Indian chewers
commence before the age of 20 years, and a negligible number commence after
40 years.

673

674               MA1tY SCHONLAND AND EVIELYN BRADSHAW

TABLE III.-Age at which Betel-Chewing Began-Chewers Aged

20+ Years

(a)                   Males            Females

Years       Hindu    Moslem   Hindu    Moslem

0-9 .    .   .    7.4      -        2-5      5B2
10-19    .    .  26*0      43*5     32*5     48.3
20-29    .    .   33-3     47-9     46*5     31*9
30-39    .    .   25-9      4*3     11.9     11*2
40-49    .    .   3*7       -        4*1      2*6
50-59    .    .    3*7      4-3      1*7      0-9
60+      .    .                      0-8      -
All ages-total .  54       23      363       116

Mean age (years)  23 9     21-7     23-8     20 2
(b)

All Indian  All Indian

males       females   All Indians
Years          %            %           %
0-19     .   .     36-4    .    394    .   39 0
20-39    .    .    571      .   54.7    .   55.1
40+      .    .     6-5     .    5.9    .    5.9
Mean age (years)   23.3     .    22 95

Frequency of betel-chewing

The number of times betel was chewed varied, some taking it very occasionally,
and others four or more times a day. This distribution has been simplified into
those who chew less than once a week (occasional chewers), those who chew between
1 and 6 times a week (light chewers), those who chew between 1-3 times a day
(moderate chewers) and those who chew betel 4 or more times a day (heavy
chewers).

When Hindu and Moslem males and females were analysed separately there
were no significant differences within the sex divisions as to chewing. When
these four groups were analysed by age there were no significant differences as to
frequency of chewing between older and younger members, and no tendency for
older chewers to chew oftener than younger chewers. When all male chewers
and all female chewers are compared there is a significant difference as to the
frequency of chewing betel. Table IV shows that the difference lies in the fact
that more females are heavy chewers and more males are light or occasional
chewers.

TABLE IV.-Frequency of Chewing-Chewers Aged 20+ Years

Indian males  Indian females
No.     %      No.     %
Occasional.   .   20    26-0     59    12-3
Light     .   .   21    27v3     75    15 7
Moderate .    .   30    39 0     221   46-1
Heavy     .   .    6     7*8    124    25 9
All chewers   .   77             479

Chi-Square:   .    : 24 - 06; P < * 01 Significant

ALIMENTARY TRACT CANCER AND BETEL CHEWING

Duration of the betel-chewing habit

The duration of the habit depends on the age of the chewers, but as the various
groups of chewers aged 20 years or more showed no significant differences in age
distributions, and were therefore matched groups, comparisons of the duration
of the habit can be undertaken. There were no differences between Hindu and
Moslem men or women. They are therefore grouped together and a comparison
is made between all Indian males and all Indian females in Table V. This table
indicates that, among chewers, there are no significant sex differences in the
duration of habit.

TABLE V.-Duration of Betel-Chewing Habit-Chewers Aged

20+ Years

Indian males  Indian females
Years of chewing    No.     %     No.     %
Less than 10 years  .  .  28   36- 3  185    38 5 6
10-19 years  .  .    .  18     23-4   126    26-3
20-29 years  .  .    .   15    19-5    83    17-3
30-39 years  .  .    .  12     15-6    45     9*4
40+ years   .   .    .   4      562    40     8*4
All chewers  .   .   .  77            479

Mean duration of chewing. 17 v 6 years  17 - 09 years

Chi-square  .    .   . X': 377;0 050 > P > 0 30Notsignificant

Total cons,umption of betel quids

The total or life-time consumption of betel-quids is the product of the years of
chewing and the number of quids chewed per day. Values were calculated which
varied from less than one to over 100. Hindus and Moslems were grouped together,
as neither the totals nor separate analyses in decennial age groups indicated that
there were significant differences between Moslems and Hindus. The total
consumption of betel-quids by Indian males and females is shown in Table VI.
The differences between Indian male and female chewers were significant, there
being fewer male chewers in the higher consumption scores and fewer females in
the lower consumption scores. As the duration of chewing was not different
between the sexes, this difference was due mainly to the greater daily frequency
of chewing in the female group.

TABLE VI. Total Consumption of Betel-Quids: Chewers Aged

20+ Years

Indian males  Indian females
Lifetime consumption  No.     %     No.     %
<1      .    .   .    .  20     26-0    50    10*4

1-9   .    .   .    .  25     32*4   136    28.4
10-19  .    .   .    .   7      9.1    48    10.0
20-49  .    .   .    .  12     15 6    99    20*7
50-99  .    .   .    .   6      7.8    64    13*4
100+   .    .   .    .   7      9.1    82    17.1
No. of chewers  .    .  77            479

Chi-suar  X :: 18 30;P < 0 01 Significant

675

Chi-square

MARY SCHONLAND AND EVELYN BRADSHAW

Preparations in use

The most usual way to prepare the betel-quid is to wrap various ingredients,
most important of which is the nut, into a fresh betel-leaf and then to place it
in the mouth and chew it. The nut is often chewed alone, and red, white or black
nuts were stated to be preferred by various chewers. By definition all betel-
chewers chewed either the leaf or the nut or both. Table VII indicates this.

There were no significant differences between males and females, or between
Moslems and Hindus as to the types of preparations in use, and there were only
slight differences in preferences for ingredients. These will be mentioned at
relevant points.

TABLE VII.-The Use of Betel-leaf and Areca Nut by Chewers

Aged 20+ Years

Males                 Females

Moslem   Hindu   Both  Moslem   Hindu   Both

Leaf, but not nut  -     7 4    5*2    5 2      2 * 2  2 * 9
Nutalone .   .  17-4    35-2   29-9   26.7     29*5   28 8
Leaf and nut  .  826    57*4   64 *9  68*1     68 *3  68* 3
Number of chewers 23    54     77     116     363    479

Female chewers differ little in their leaf and nut preferences; male Moslems
chew the nut alone less frequently than other groups. The differences were not
significant. It is interesting to observe that almost 30 % of males and females
prefer to chew the nut only. Age breakdowns indicate that younger people
prefer the nut only.

Innocuous ingredients which are sometimes added for the purpose of flavouring
the quid include spices, seeds, flavourings and nuts, including cocoanut.

Three other ingredients (lime, tobacco, and catechu) are also added to the basic
leaf/nut choice, and these may not be innocuous:

Lime is a white creamy substance, which is strongly alkaline, and a small
smear of this is added to the nut or the leaf package. Lime is added by 63.4%
of females and 64-9 % of males, Moslems and Hindus differing little.

Tobacco is added as a small quantity of coarse shreds. Although frequently
added in India, in Durban only 7.8% of male chewers and 2.8% of female chewers
add tobacco. Although the number of tobacco-users was small, it appeared to
be used more often by Hindu males and Moslem females.

Catechu is a coarsely powdered plant gum, imported from India. It is added
by 32-5% of male chewers and in 14-2% of female chewers, being used much
oftener by Moslems and Hindus.

Both tobacco and catechu were more frequently used by the older chewers
than the younger chewers, and the habit of adding these ingredients may be dying
out in Durban. A full list of the various combinations used by betel-chewers is
given in the Appendix Table C and the ingredients commonly used are dealt with
in the discussion.

Evidence suggests that the betel-chewing habit is addictive, in the same way
that tobacco-smoking is. Abstinence, for whatever reason, may cause anxiety,
and in some people withdrawal symptoms similar to those seen at times with
tobacco addicts.

676

ALIMENTARY TRACT CANCER AND BETEL CHEWING

Reasons for chewing betel

Chewers over 20 years old gave their reasons for chewing the betel package.
These were not very well formulated by the chewers and some replies (e.g. " to
pass the time ", " when I have nothing else to do ") were difficult to classify.
Reasons fell into 4 main groups: Social (" I chew at weddings", " I take it when
offered "), Habit (" I learnt to chew from my parents ", " If I don't I feel odd ",
" I cannot stay without it "), Health (" It helps my digestion ", " if I don't I
feel giddy ", " it helps me when I am pregnant not to feel bilious ") and Pleasure
(" I like the taste ", " I do it for fun "). Many chewers gave two or more reasons.
Health reasons were given by 43 % of women and 31% of men, Social reasons by
18 % of women and 27 % of men, Habit was given as the reason by 40 % of women
and 22% of men, and Pleasure was given by 26% of women and 35 % of men.
Every chewer proffered some sort of reason for chewing, and it is interesting to
note that a larger number of female chewers considered it to be good for health
than considered it to be pleasurable.

Twenty per cent of chewers (16 men and 105 women) had abandoned the
habit or cut down on it. Economy and inaccessibility were given as reasons by
16% of these, reasons of health, including adverse physical and psychiatric effects
were given by 60%, and dislike of the habit by 11%. Thus health reasons were
also frequently used to explain cessation or diminution of the habit.
Effects on chewers, pleasant and unpleasant

In an attempt to ascertain whether the betel habit had any pleasant effect
on mood, questions as to soothing and stimulating effects were asked: 39% of
men and 32% of women said it had no effect; 40% of men and 30 % of women
said it had a soothing, calming effect; 9 % of men and 12% of women said it had a
stimulating effect; 12 % of men and 25 % of women said it had both a soothing and
stimulating effect. Thus the effects, if felt, were tranquillising on the whole.

Many chewers reported that there were unpleasant effects relating to the habit.
Complaints were given in detail by the chewers, and there were often several com-
plaints by one chewer.

Staining of mouth or teeth, the commonest complaint, is caused by juices of
the areca nut; the sialogogic effect of the betel package is very marked, but the
authors are not certain whether the nut, leaf or lime is responsible. A diaphoretic
effect and a feeling of being giddy or drugged noted by some chewers suggests that
elements of the betel package have some systemic effect after absorption.
Although not many chewers complained of discomfort felt in the mouth, betel-
chewing has been implicated in the causation of submucous fibrosis of the buccal
cavity and elements of the quid may have a direct local effect on the buccal
mucosa, either chemical or traumatic (Shear et al., 1966; Pindborg, 1965).

DISCUSSION

It has been established that Indian females in Natal have higher cancer inci-
dence rates for the mouth, pharynx, oesophagus and stomach than men, and that
these rates are higher than those for English people. It has also been established
that a far greater percentage of Natal Indian women chew betel than men and that
among chewers, women chew more heavily than men. These differences are
significant.

An association between poor oral hygiene, oral submucous fibrosis, leukoplakia

55

677

MARY SCHONLAND AND EVELYN BRADSHAW

and oropharyngeal cancer has been noted by workers in Asia (Paymaster, 1956
and 1962; Pindborg, 1965; Pindborg et al., 1967; Wahi et al., 1965). Oral submu-
cous fibrosis, first described by Joshi in 1953, was considered to be caused by the
betel chewing habit by some observers (Shear et al., 1966; Paymaster, 1962;
Pindborg, 1965; Pindborg et al., 1967) but not by others (Tennekoon and Bartlett,
1969; Balendra, 1949; Khanolkar, 1944). All workers agree that oropharyngeal
cancers are common in countries where betel-chewing is a habit. This habit
has been well described by Muir and Kirk (1960) and others (Orr, 1933; Davidson,
1923; Hirayama, 1966). Most workers have noted that tobacco is the important
ingredient in the betel-chewing habit, and many have observed that dietary defici-
encies, in particular Vitamin A deficiencies, are also present in the betel-chewing
population (Paymaster, 1956; Wahi et al., 1965; Orr, 1933). Most workers suspect
tobacco as being causative both of submucous fibrosis and oropharyngeal cancer.
Hirayama (1966) and Orr (1933) regard the lime component as being important
in liberating carcinogenic alkaloids from tobacco. Hitherto almost all workers
have disregarded the leaf and the areca nut as carcinogenic hazards.

As there is an association in Natal Indians between betel-chewing and upper
alimentary tract cancer, but very little usage of tobacco in the quid, it becomes
apparent that the leaf, the nut and the lime components must be reconsidered
as possible carcinogens.

It is unlikely that poor oral hygiene, deficient diets, and curry or chilli consump-
tion, which have been suggested to be cancer-promoting by several workers
(Paymaster, 1956; Pindborg et al., 1967; Wahi et al., 1965; Balendra, 1949) are
relevant, because these elements are equally present between males and females
in Natal, and yet the incidence of upper alimentary tract cancer is not.

Muir and Kirk (1960), Orr (1933) and Davidson (1923) have given careful
descriptions of the leaf, the nut and lime, as follows:

(a) The leaf: Mature leaves of the betel vine (piper betle) contains volatile
oils (eugenol, an unsaturated aromatic phenol, and terpenes) potassium nitrate,
and small quantities of sugar, starch and tannin. It has a carminative effect,
sweetens the breath, and is a gentle stimulant. Natal chewers use betel leaves
grown on special farms to the north of Durban. (Indian name: paan.)

(b) The nut: The dried areca nut contains many alkaloids (arecoline, arecaine,
guvacaine, arecolidine, guvacoline, iso-guvacine and choline) chief among which
is arecoline, which has a muscarinic (cholinergic) effect (Pffeiffer et al., 1967) which
is glandular secreting, gut-stimulating, vasodepressant, and has a direct cortical
arousal effect. Muir and Kirk (1960) state that it is sialogogic and diaphoretic,
and that very large amounts depress the central nervous system. It has been
used in Malaya as a vermifuge and as a cure for diarrhoea. Pfeiffer et al. (1967)
state that it may be used to counteract schizophrenia, and inhibits conditioned
responses in laboratory animals. It is likely that the soothing, stimulating, and
diaphoretic effects reported by Natal chewers are due to the nut alkaloids, and the
frequently made claims by chewers that it is good for digestion may be an expres-
sion of the carminative effect. Arecoline is probably absorbed in quite large
amounts by regular chewers as 0-1 % of the dried nut is arecoline. In addition
the nut also contains tannin, fatty acid glycerides and sugar. Natal users import
the nut, which has three main varieties, the red, the white and the black. These
are probably different species of the areca nut, the black being the driest and
hardest. (Indian name: supari.)

678

ALIMENTARY TRACT CANCER AND BETEL CHEWING

(c) Limee: In India two types of lime may be used. The commoner type is
prepared from limestone, but near the coast, lime is made from shells and is
finer in consistency. Orr (1933) considered lime to be an injurous ingredient in
betel-chewing, and shell-lime more so than stone-lime. He stated that lime cata-
lysed the liberation of carcinogenic alkaloids from tobacco of the quid. Hirayama
(1966) found that oral cancer occurred where tobacco and lime were chewed
together, but not when tobacco was chewed alone, and felt that very strong
suspicion should be attached to lime either as a carcinogen or as carcinogen-
liberator from tobacco. Muir and Kirk (1960) felt that lime, in the small quanti-
ties used, served merely to neutralise the acid taste of the nut. Tennekoon and
Bartlett (1969) felt that it might have an irritant action, but was used in such
small quantities, that dilution by saliva rendered it innocuous. In Natal, the
action of lime on tobacco is not in question, and the quantity added to the quid
is very small. It is therefore difficult to say whether the lime is a suspect ingredi-
ent, either by its own action, or as a catalyst for releasing possibly carcinogenic
alkaloids from the areca nut. Natal chewers obtain the lime from limestone,
from a local supply source. (Indian name: chunam.)

(d) Catechu: This substance is extracted from the leaves of the shrub uncaria
gambir. The leaves are bound, steamed, and steeped in boiling water. On
cooling, catechin crystallises out, leaving the more soluble catechu-tannic acid in
solution. Bran is added to catechin, and then made into little cakes. In Malaya,
gums from acacia species are used instead. Natal users import the catcchu
cakes. (Indian name: gettah gambir, or katta kambu.)

(e) Tobacco: In Natal a dried leaf grown in the Transvaal is used. It is coarsely
shredded and a few flakes are added by the small proportion of chewers who use it.
In India, various types of tobacco are used, and various methods of curing have
been described which differ from the local practice.

The authors feel that the areca nut, and possibly the leaf and lime, being
so commonly used in Natal, are the most suspect ingredients of the betel-chewing
habit in Natal. We do not consider catechu or tobacco to be hazards, as so few
people add these and because their use is losing popularity among younger
chewers.

Very little experimental work has been carried out on areca nut, betel-leaf
or lime, as potential carcinogens. Muir and Kirk (1960) and Tennekoon and
Bartlett (1969) have summarised the evidence, most of which has been based on
betel ingredients plus tobacco.

Shear et al. (1966) observed that betel-chewing was more prevalent among
patients with submucous fibrosis than among the general South African Indian
population. They also observed that oral submucous fibrosis was very rare in
Indian males; the condition was not observed at all in non-Indian South Africans.

SUMMARY

The general features of the South African Indian community are outlined.
A recent survey of cancer morbidity incidence in Durban Indians indicates that
cancers of the mouth, throat, oesophagus and stomach are commoner in Indian
females than males, and that the combined incidence of these neoplasms is high.
Realisation that the habit of betel-chewing in Durban Indians was also predom-
inantly a female habit promoted an intensive study of the habit among Durban
Indians. Analysis of this study shows that 8-3% of Indian males and 54-2% of

679

680             MARY SCHONLAND AND EVELYN BRADSHAW

Indian females aged 20 years or more are addicted to betel-chewing, and this
difference is significant. Schooling attainments and ability to speak English,
when regarded as indices of westernisation, showed that younger people of both
sexes were more westernised, and that males at all ages were more westernised
than females. Differences between Moslems and Hindus (age-adjusted) were not
significant. The tendency towards greater westernisation in Indian males may
be the main reason for less betel-chewing in the male group, and it is likely that
the habit in both sexes may be dying out.

Among chewers, comparisons between males and females (age-matched groups)
showed that the betel-quid is chewed significantly more frequently by females,
and that the lifetime consumption of the female group was significantly higher than
that of the males. Duration of the habit, and age of adoption of the habit did
not differ between the sexes. Moslem and Hindu differences were not significant,
but there was a tendency for Moslems to adopt the habit at an earlier age.

The commonest ingredients used in the betel-quid are the leaf, the areca nut
and the slaked lime. Tobacco is used very rarely. Reasons for adoption of the
habit are most commonly given as being for social or health reasons. Unpleasant
effects include a diaphoretic effect. Pleasant effects seem to be predominantly
tranquillising.

This study differs from previous studies made by workers in India and the
East in that tobacco is seldom added as an ingredient in Natal, and in that sex-
differences in cancer of the upper alimentary tract and in the prevalence of the
betel-chewing habit are marked, both being commoner in females. This observa-
tion must direct our attention to a possible association between betel-chewing and
upper alimentary tract cancer, and to elements other than tobacco in the betel-
quid which may be cancer promoting. The leaf, nut, and the lime component
should in the first instance be investigated.

This study was financed by the National Cancer Association of South Africa,
and we are indebted to the Institute of Social Research, Natal University, for
assistance and advice.

REFERENCES

BALENDRA, W.-(1949) J. Ceylon Brch Br. med. Ass., 38, 47.
DAVIDSON, J.-(1923) Br. med. J., ii, 733.

HIRAYAMA, T.-(1966) Bull. Wld Hlth Org., 34, 41.
JOSHI, S. G.-(1953) Ind. J. Otolar., 4, 1.

KHANOLKAR, V. R.-(1944) Cancer Res., 4, 313.

MCCRYSTAL, L. P. and MAASDORP, G. G. (1967) Paper presented at Conference on "The

Indian South African", South African Institute of Race Relations.
Mum, C. S. and KiRK, R.-(1960) Br. J. Cancer, 14, 597.
ORR, I. M.-(1933) Lancet, ii, 575.

PAYMASTER, J. C.-(1962) Cancer, N.Y., 15, 578.-(1956) Cancer, N.Y., 9, 431.
PINDBORG, J. J.-(1965) Bull. Wld Hlth Org., 32, 749.

PINDBORG, J. J., POULSEN, H. E. and ZACHARIAH, J.-(1967) Cancer, N. Y., 20, 1141.

PFFEIFFER, C. C., BECK, R. A. and GOLDSTEIN, L.-(1967) Ann. N. Y. Acad. Sci., 142,181.
POPUILATION CENsus-(1960) Bureau of Statistics. Pretoria (Government Printer).
SCHONLAND, M. and BRADSHAW, E.-(1968) Int. J. Cancer, 3, 304.

SHrEAR, M., LEMMER, J. and DOCRAT, I. S.-(1966) Med. Proc., 12, 541.
TENNEKOON, G. E. and BARTLETT, G. C.-(1969) Br. J. Cancer, 23, 39.

WAHI, P. N., KEHAR, U. and LAuini, B.-(1965) Br. J. Cancer, 19, 642.

ALIMENTARY TRACT CANCER AND BETEL CHEWING

APPENDIX

TABLE A.-Betel-Chewing in Durban Indians: Age, Schooling, and

Ability to Speak English Allied to Sex

Males                          Females

No. of     No. of   Chewers     No. of     No. of    Chewers
people    chewers       %       people    chewers       %
406         10         2-5   .   425         19        4.5
497         14         2-8   .   534         67       12 5
401         16         4 0   .   359        125       34-8
194         21       10-8    .   219        139      63-5
154         15        9 7    .   154        107       69-5
106         17       16 0    .    94         67       71 3

78          8       10 3    .    57         41      71 9
1836        101         5-5   .  1842        565       30 7
903         24         2-7   .   959         86        9 0
933         77         8-3   .   883        479       54-2

Schooling: 20 years and over

Nil     .    .    67
Up to Std. 2  .   75
Stds. 3-6    .   531
Std. 7 and over  260

933

10

9
43
15

77

14 9
12-0

8*1
5 8
8*3

English spoken: 20 years and over

Nil               78        12       15 3
Littlef.7                    2       1~

Fluent.      .   855        65        7-6
Total   .    .   933        77        8 3

328
114
352

89
883

70
240
573
883

231

80
146

22

479

52
181
246

479

TABLE B.-Education and Abi

and Sex: Indians Aged 20+

MALES

Education

Nil

Up to Std. 2.
Stds. 3-6

Std. 7 or more

English spoken

Nil or little
Fluent

FEMALES
Education

Nil

Up to Std. 2.
Std. 3-6

Std. 7 or more

English spoken

Nil or little
Fluent

20-29

1
2
58
39
100

2  .   5
89 . 95
100 . 100

12
. . 10

59
19

100

10
90
100

35
17
40

8
100

35
65

100

ility to Speak English Allied to Age
Years, Expressedi as Percentages

40-49 50-59 60+ Total 20-60+

6  . 17   . 41   .      7
12  . 22   . 15   .      8
63  . 53   . 39   .     57
19  .   8  .   5  .     28
.100   .100   .100   .   100

. 10
* 90
. 100

52
21
25

2
. 100

. 50

50

. 100

17
83
100

83

5
12

100

79
21

100

. 36
. 64

. 100

91

5
4

. 100

82
18

. 100

8
92

100

37
13
40
10

100

35
65

100

* Percentages correct to nearest unit.

681

Age
0-9
10-19
20-29
30-39
40-49
50-59
60+
Total

Under 20

20 and over

Total

70 4
70-2
41-5
24- 7
54*2

74.3
75.4
42 9

54 2

MARY SCHONLAND AND EVELYN BRADSHAW

TABLE C.-Ingredients Used in Betel-Quid (Male and Femawle

Chewers Aged 20+ Years)

All male    All female
chewers     chewers

No.   %      No.    %
Nut only  .    .    .    .    . 23    29 9  . 131   27 3
Nut + lime     .    .    .    .             .   7    1P5
Leaf only  .   .    .    .    .   1    1-3  .   7    15
Leaf + catechu .    .    .    .   1    1-3

Leaf + lime    .    .    .    .   2    2-6  .   6    1-3
Leaf + nut     .    .    .    .   3    3 9  . 30     6 3
Leaf, nut, lime  .  .    .    . 23    29 9  . 228   47- 6
Leaf, nut, catechu  .    .    .   3    3 9  .   7    15
Leaf, nut, lime, catechu  .   . 15    19 5  . 49    10-2
Leaf, nut, lime, tobacco  .   .   2    2 - 6    3    0 6
Leaf, nut catechu, tobacco  .  . -          .   1    0 2
Leaf, nut, lime, catechu, tobacco  .  4  5 2  . 10   2 0

Total     .    .    .    .    . 77   1001   . 479  100 0

No. % of 77   No. %of479
Leaf, (ever)   .    .    .    . 54    701   . 341   71P2
Nut, (ever)    .    .    .    . 73    94-8  . 465   97-1
Lime (ever)    .    .    .    . 46    59 7  . 304   63-5
Catechu, (ever) .   .    .    . 23    29 9  . 68    14-2
Tobacco, (ever) .   .    .    .   6    7 8  . 14     2 9
Seeds, spices, nuts, honey, (ever)  . 10  13-0  . 54  11 3

682

				


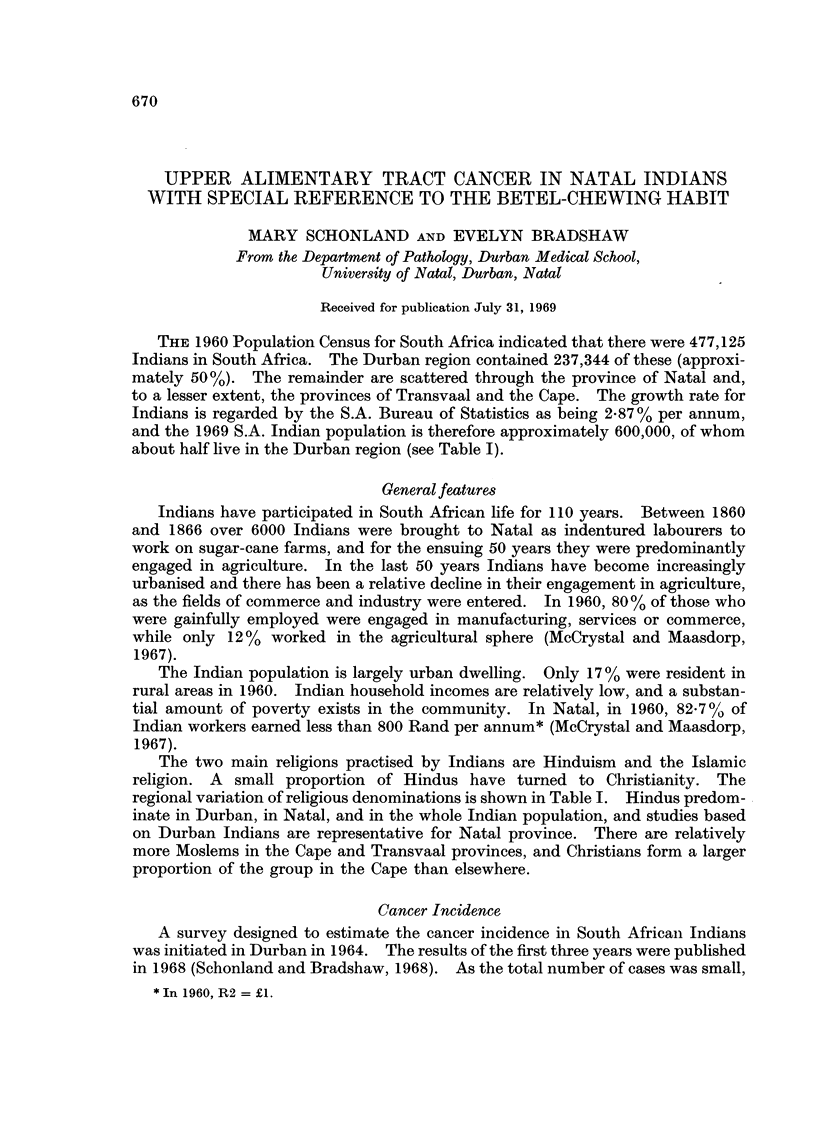

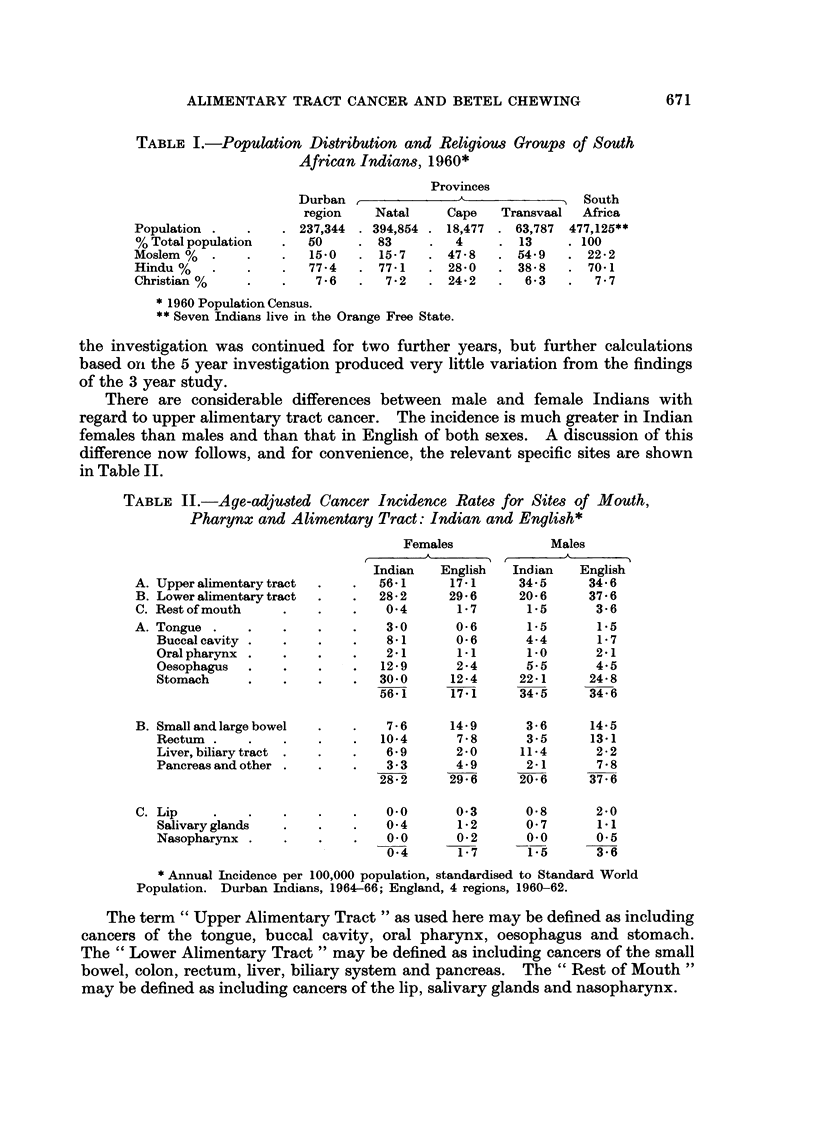

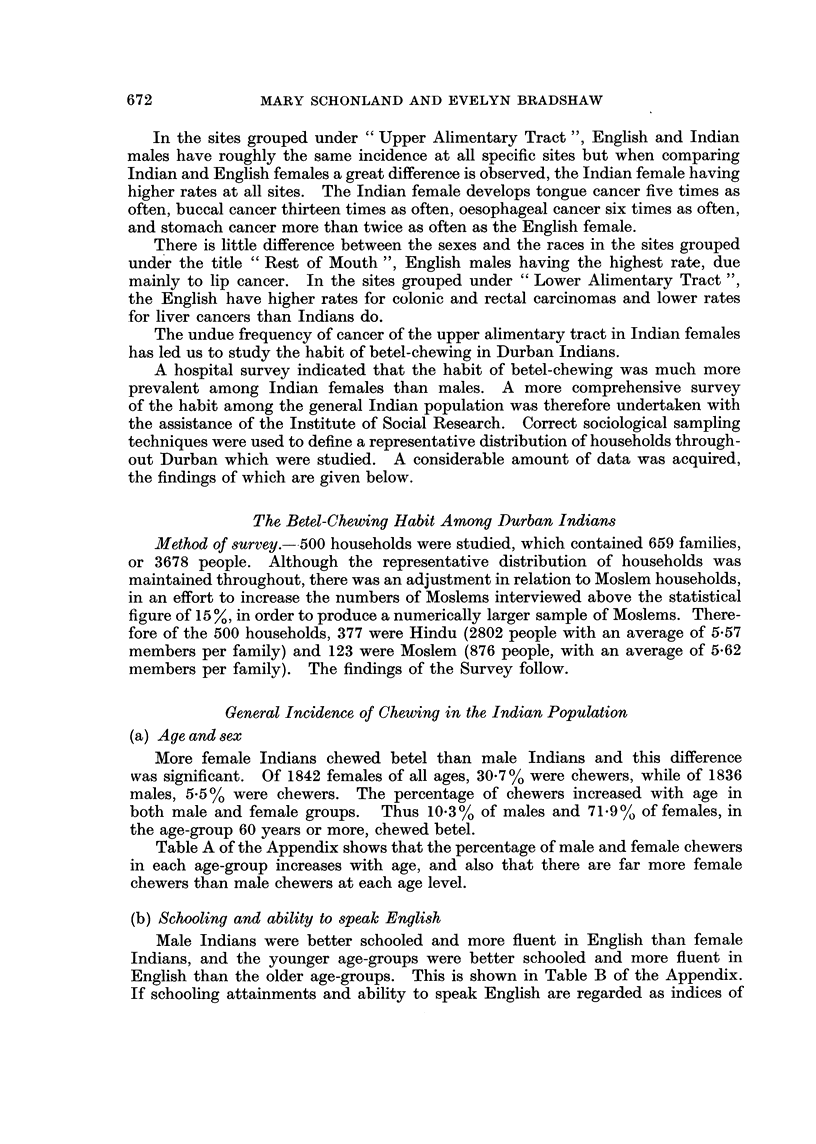

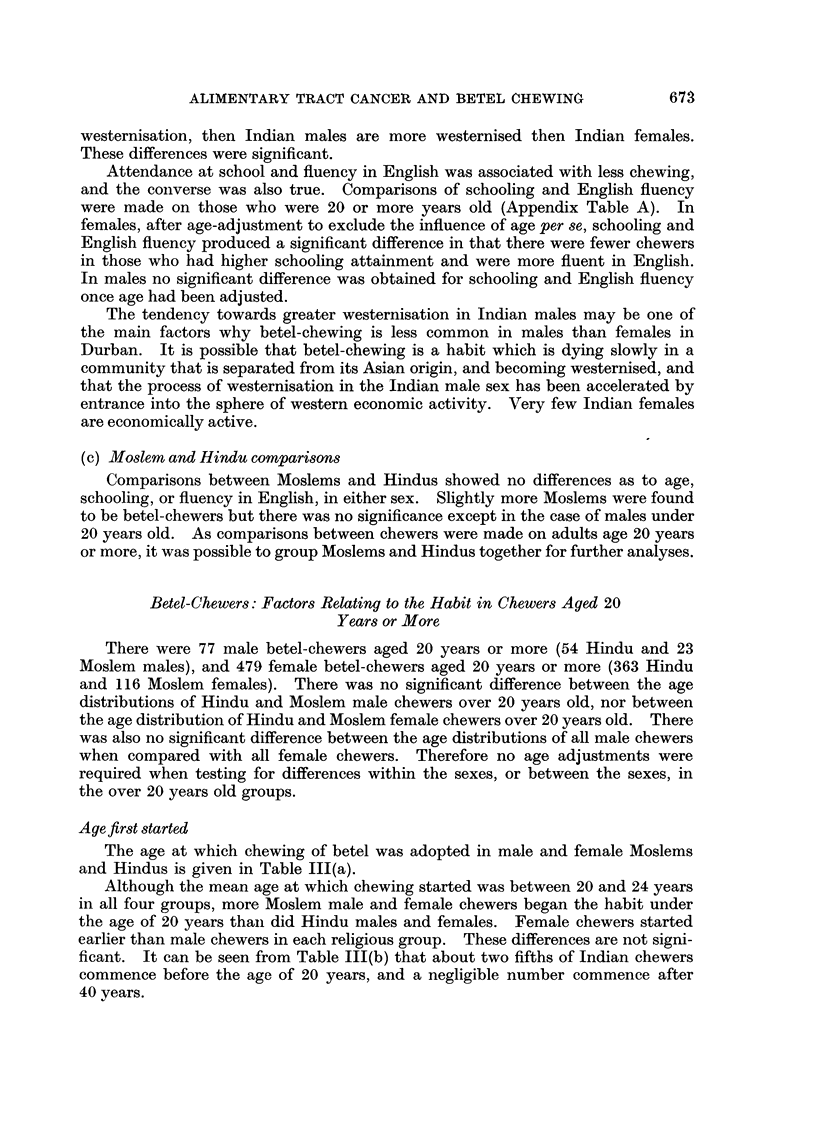

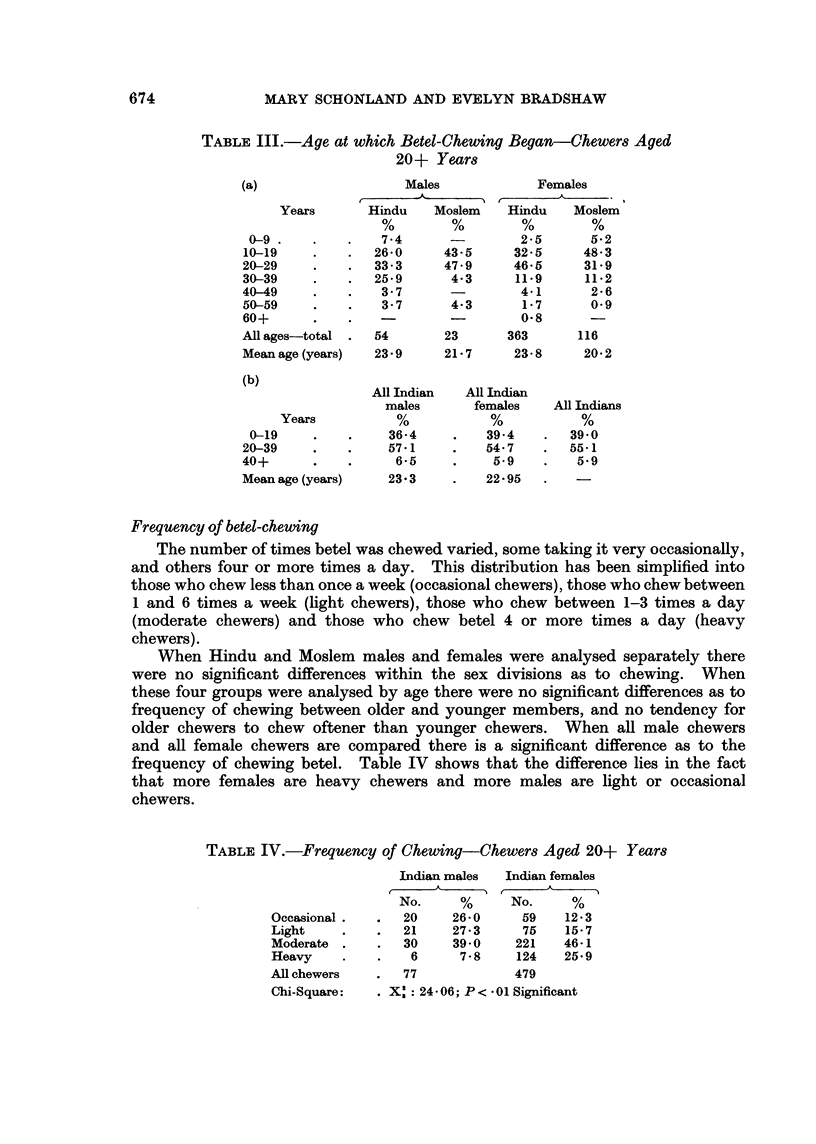

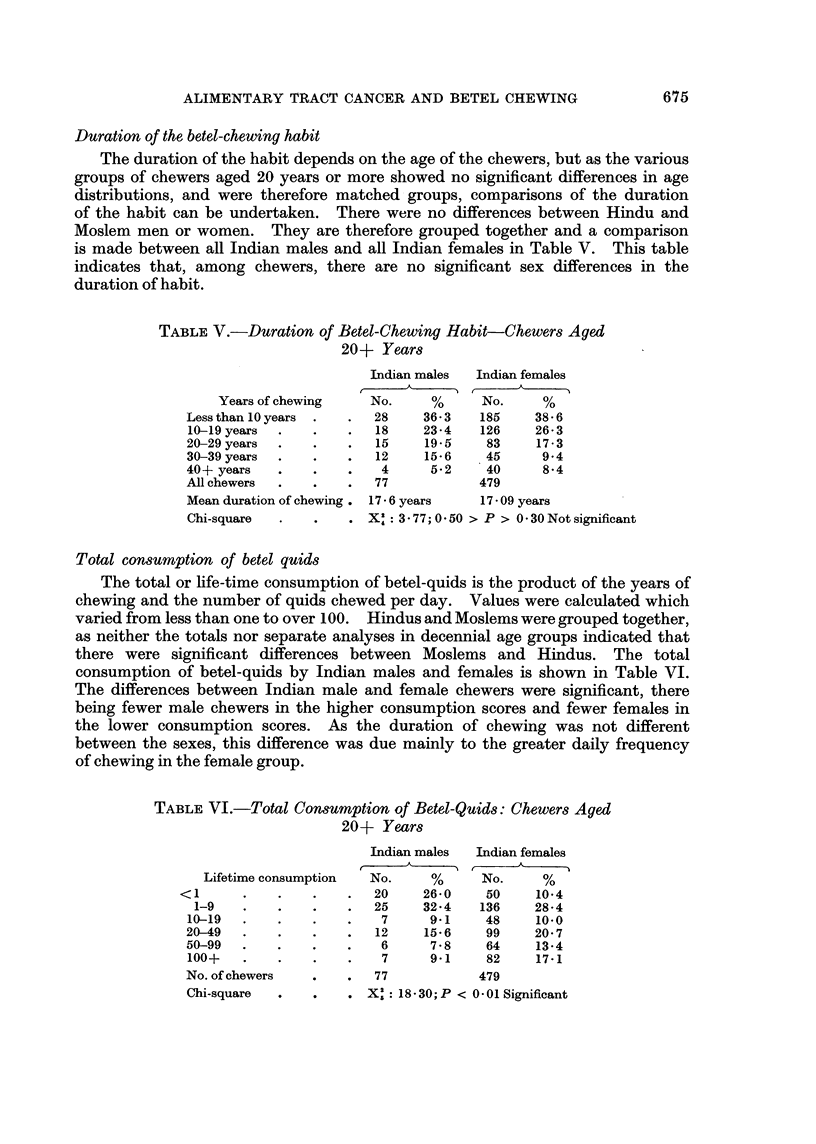

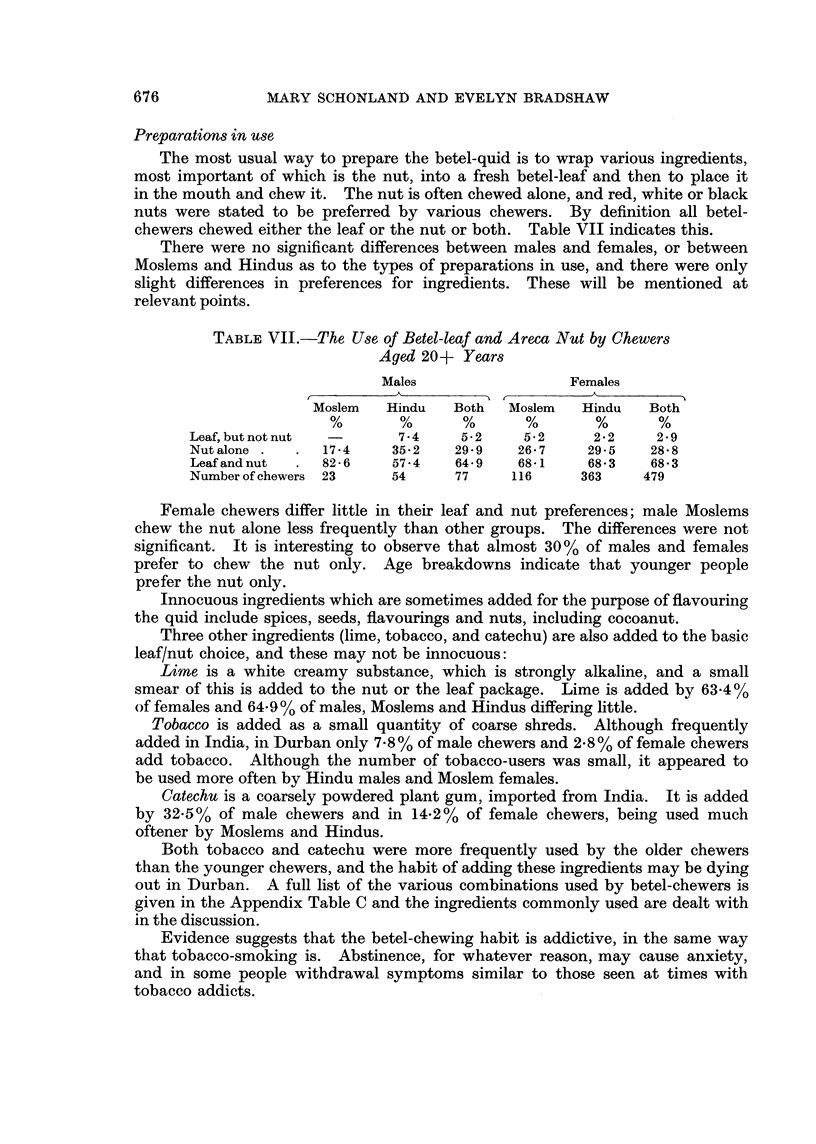

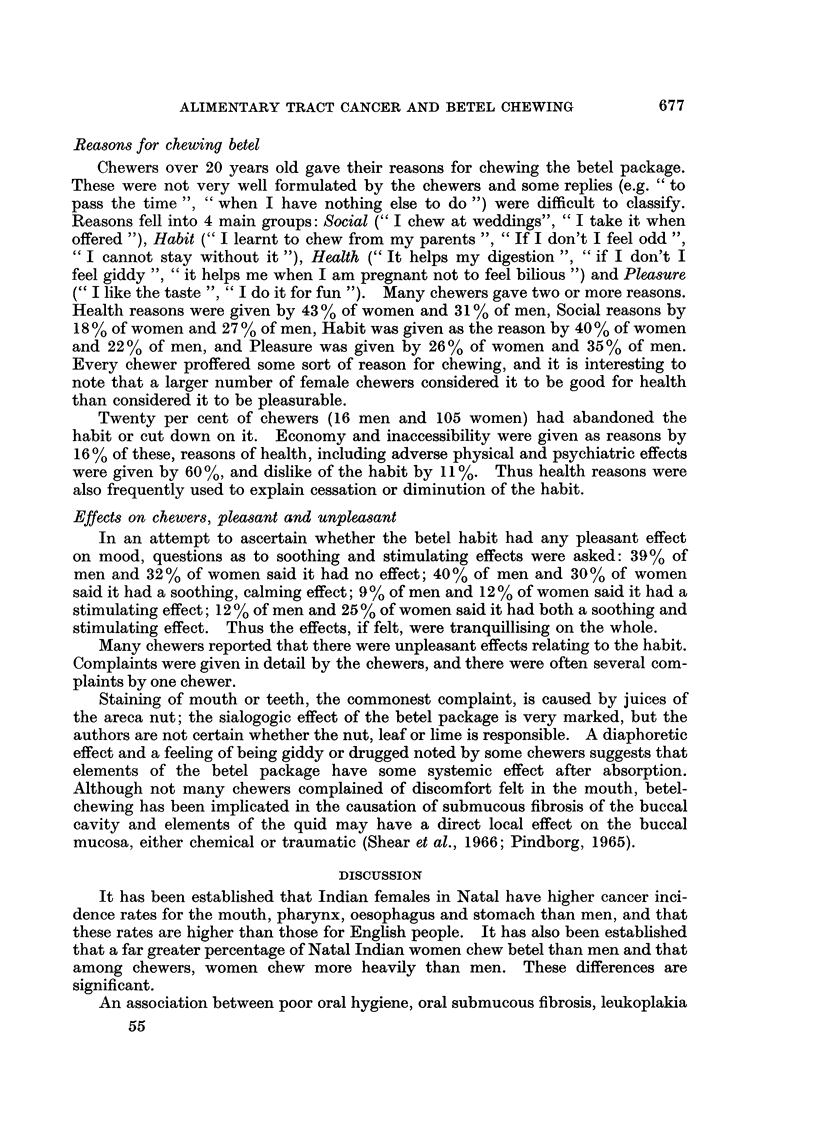

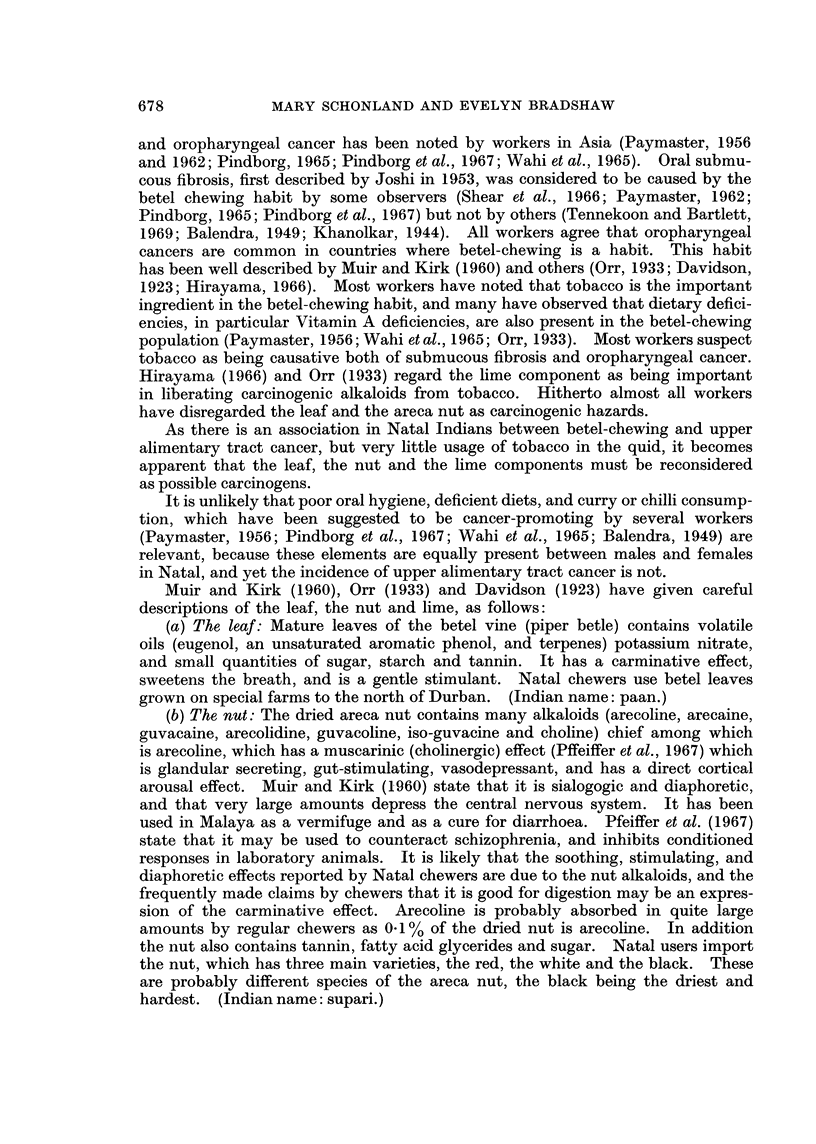

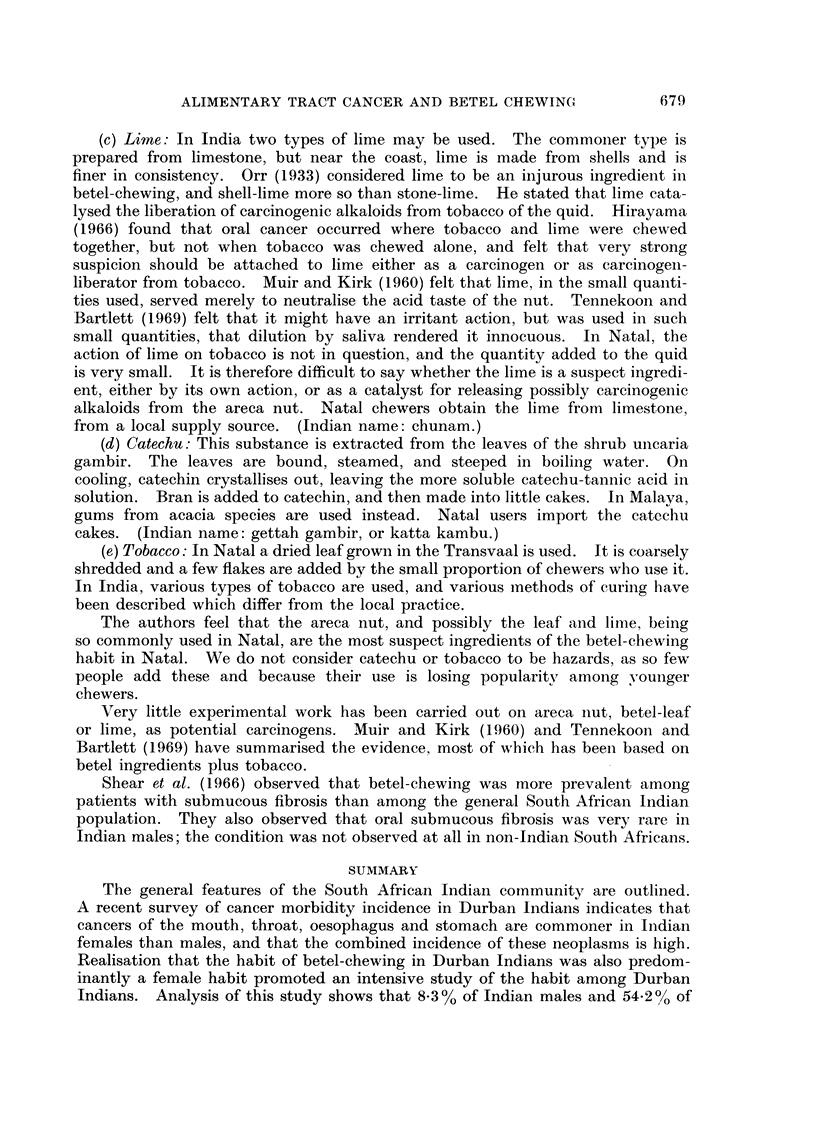

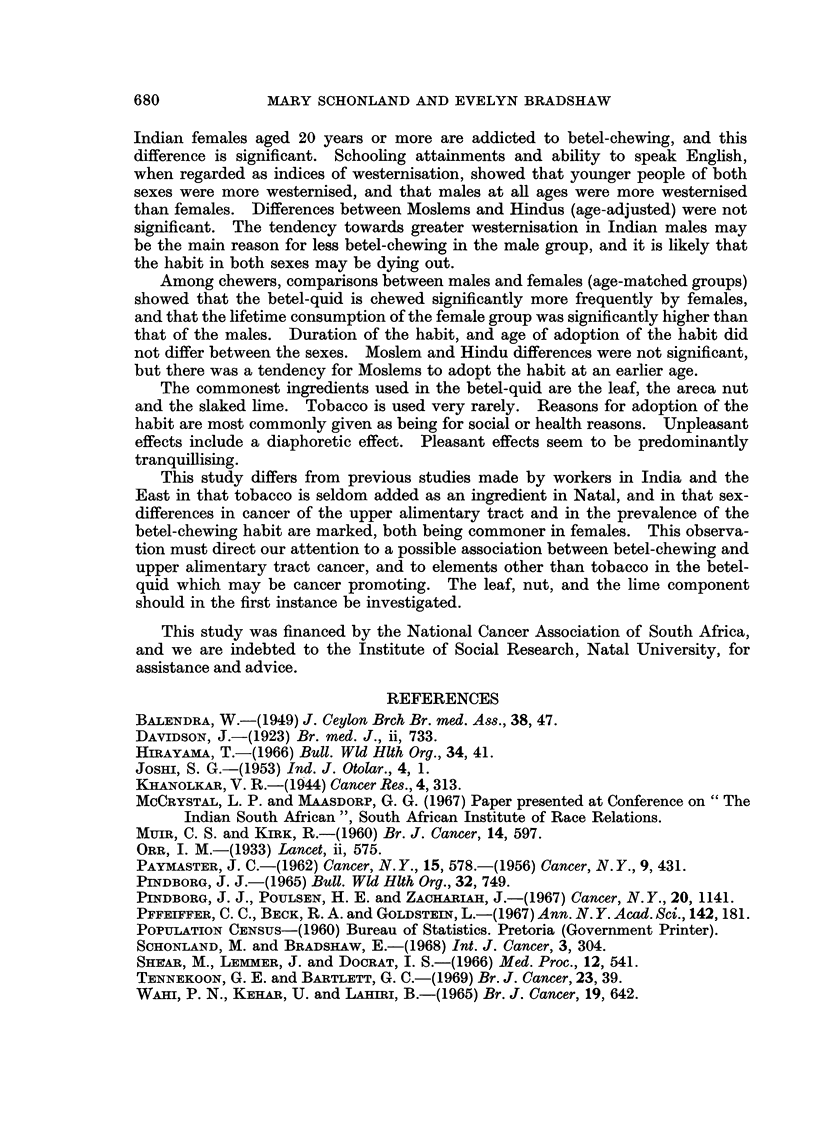

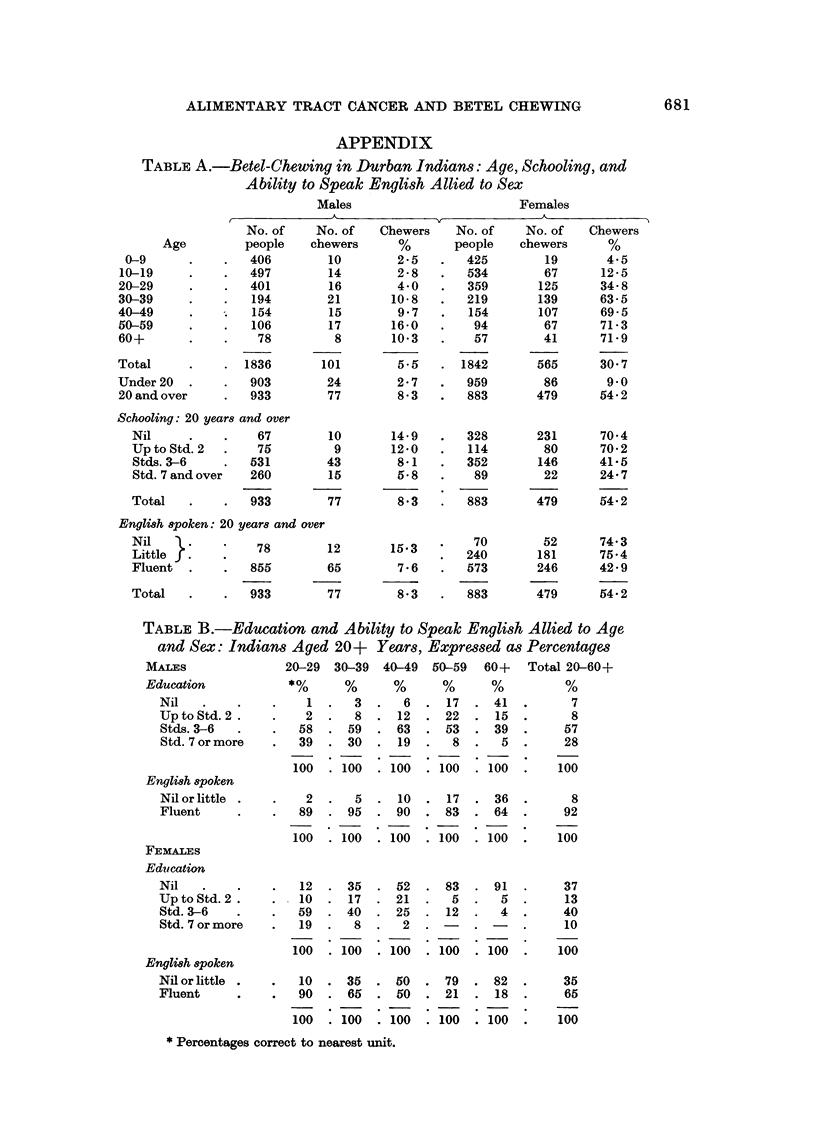

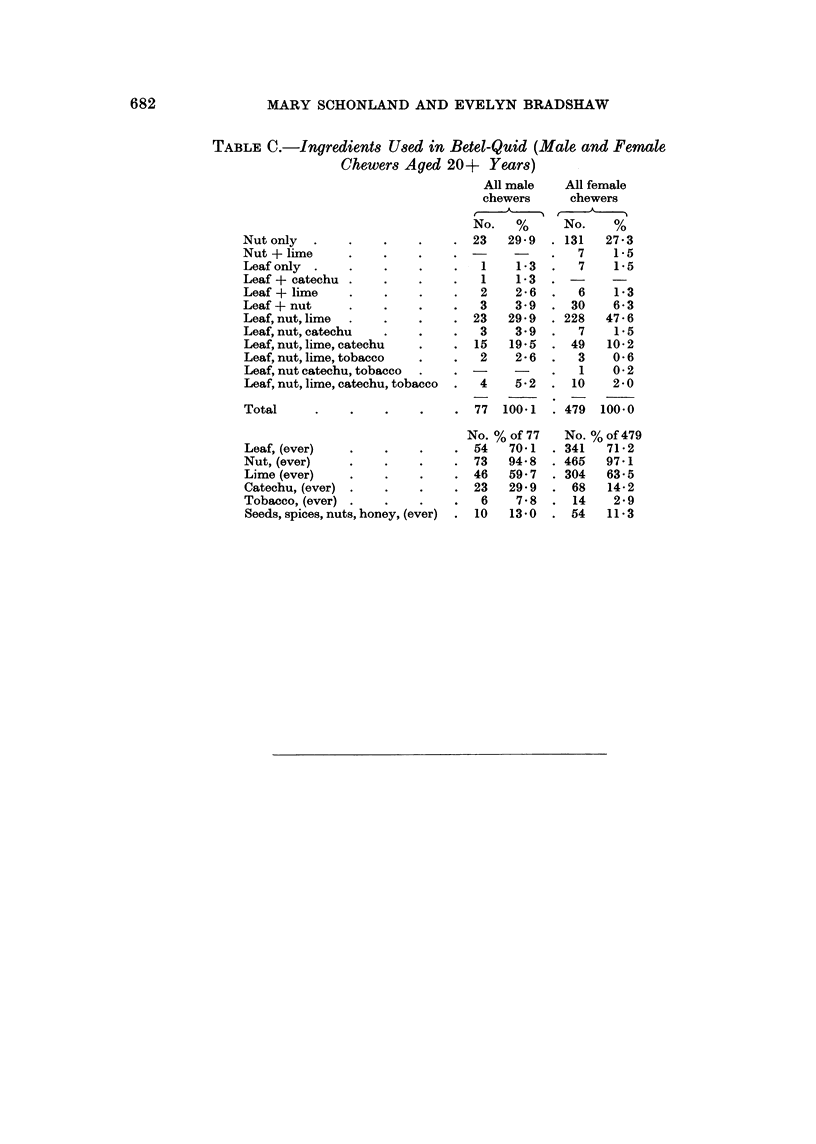

